# LZAP Inhibits p38 MAPK (p38) Phosphorylation and Activity by Facilitating p38 Association with the Wild-Type p53 Induced Phosphatase 1 (WIP1)

**DOI:** 10.1371/journal.pone.0016427

**Published:** 2011-01-24

**Authors:** Hanbing An, Xinyuan Lu, Dan Liu, Wendell G. Yarbrough

**Affiliations:** 1 Department of Otolaryngology, Vanderbilt University, Nashville, Tennessee, United States of America; 2 Department of Cancer Biology, Vanderbilt University, Nashville, Tennessee, United States of America; 3 Barry Baker Laboratory for Head and Neck Oncology, Vanderbilt University, Nashville, Tennessee, United States of America; 4 Vanderbilt Ingram Cancer Center, Vanderbilt University, Nashville, Tennessee, United States of America; Texas A&M University, United States

## Abstract

LZAP (Cdk5rap3, C53) is a putative tumor suppressor that inhibits RelA, Chk1 and Chk2 and activates p53. LZAP is lost in a portion of human head and neck squamous cell carcinoma and experimental loss of LZAP expression is associated with enhanced invasion, xenograft tumor growth and angiogenesis. p38 MAPK can increase or decrease proliferation and cell death depending on cellular context. LZAP has no known enzymatic activity, implying that its biological functions are likely mediated by its protein-protein interactions. To gain further insight into LZAP activities, we searched for LZAP-associated proteins (LAPs). Here we show that the LZAP binds p38, alters p38 cellular localization, and inhibits basal and cytokine-stimulated p38 activity. Expression of LZAP inhibits p38 phosphorylation in a dose-dependent fashion while loss of LZAP enhances phosphorylation and activation with resultant phosphorylation of p38 downstream targets. Mechanistically, the ability of LZAP to alter p38 phosphorylation depended, at least partially, on the p38 phosphatase, Wip1. Expression of LZAP increased both LZAP and Wip1 binding to p38. Taken together, these data suggest that LZAP activity includes inhibition of p38 phosphorylation and activation.

## Introduction

LZAP (Cdk5rap3, C53) was originally identified as a binding partner of the Cdk5 activator p35 [1], but insight into LZAP activity was gained when it was found to bind the alternate reading frame protein of the INK4a gene locus, ARF (p14^ARF^ in human and p19^ARF^ in mice) and activate p53, both in the presence and absence of ARF, resulting in a G1 cell cycle arrest and inhibition of clonogenic growth [2]. Further, LZAP inhibits cellular transformation, xenograft tumor growth, and xenograft tumor vascularity at least partially through LZAP's ability to bind and inhibit RelA [3]. Evidence of a tumor suppressor-like role for LZAP was bolstered when LZAP protein levels were found to be markedly decreased in head neck squamous cell carcinoma (HNSCC) where its loss inversely correlates with expression of NF-κB target genes [3]. LZAP also inhibits the checkpoint kinases (Chk1 and Chk2), promotes mitotic entry and, in the presence of DNA damaging agents, sensitizes to cell death [4], [5]. Further exploration of LZAP regulation found that a binding partner of LZAP, RCAD/NLBP, stabilizes LZAP protein levels and loss of RCAD/NLBP results in loss of LZAP with enhanced NF-κB signaling and cell invasion [6], [7]. Collectively, these data are consistent with a role for LZAP in tumor suppression.

p38MAPK belongs to a family of stress-activated MAPKs that responds to cellular stress and cytokines. Expression patterns suggest that p38α may be the primary p38 kinase in most cell types [8]. Activity of p38 reflects a balance between the upstream activating kinases (MKK3 and MKK6) and inactivating protein phosphatases, primarily the wild-type p53-induced phosphatase 1 (Wip1, PPM1D, PP2Cδ) [9]–[10]. p38 activity results in pleiotropic downstream cellular and tissue effects including: cytokine production, inflammation, cellular differentiation, cell-cycle arrest, apoptosis, and senescence [8], [11], [12], [13], [14], [15]. Given the roles of p38 as an inducer of apoptosis and inhibitor of cellular proliferation, it is ironic that elevated p38 expression has been found in many cancer types, including breast, lung, thyroid and HNSCC, and that p38 has been implicated in promoting cell survival [12], [16], [17], [18], [19]. Given the conflicting cellular effects that can result from p38 activation, the role of p38 in human cancer as a tumor promoter or a tumor suppressor likely depends on tumor and cell specific context [8].

Here, we describe that the putative tumor suppressor LZAP bound and inhibited p38MAPK. Conversely, depletion of LZAP enhanced phosphorylation and activity of p38. LZAP did not alter p38 activating kinases (MKKs); however, LZAP increased association of p38 with Wip1 and LZAP dependent inhibition of p38 phosphorylation was at least partially dependent on Wip1. Given that LZAP inhibits p38 activity and that the role of p38 in cancers can vary from growth inhibitory to growth promoting, results presented here suggest that LZAP activities in tumors may be complex.

## Results

### LZAP interacts with p38 MAPK in *vivo*

Described LZAP activities include inhibition of cellular proliferation, inhibition of anchorage-independent growth, and enhancement of response to chemotherapeutic agents [2], [4], [5]. LZAP expression is lost in a subset of human HNSCC and loss of LZAP in xenograft tumors enhances tumor growth and angiogenesis [3]. These data suggest that LZAP may function as a tumor suppressor. Intriguingly, morpholino directed loss of LZAP expression in zebrafish resulted in cell death and developmental delay (data not shown). Combined, these data suggest that either increased or decreased LZAP levels may have detrimental effects on cell survival. To identify proteins that may contribute to biological activities of LZAP, we screened human LZAP amino acid sequence for motifs recognized by modular signaling domains using the Scansite algorithm [20]. Using high stringency criteria, Scansite analyses suggested that LZAP contained motifs predicted to bind 14-3-3-zeta and the docking domain (D domain) of mitogen activated protein kinases (MAPKs) (Table S1). Because D domain-dependent interactions are essential for MAPK binding to upstream regulators and downstream mediators [21] and because p38 and LZAP have been shown to activate p53 and to interact physically or functionally with Chk1, p38 was chosen for further exploration [12] [22] [2].

**Figure 1:**
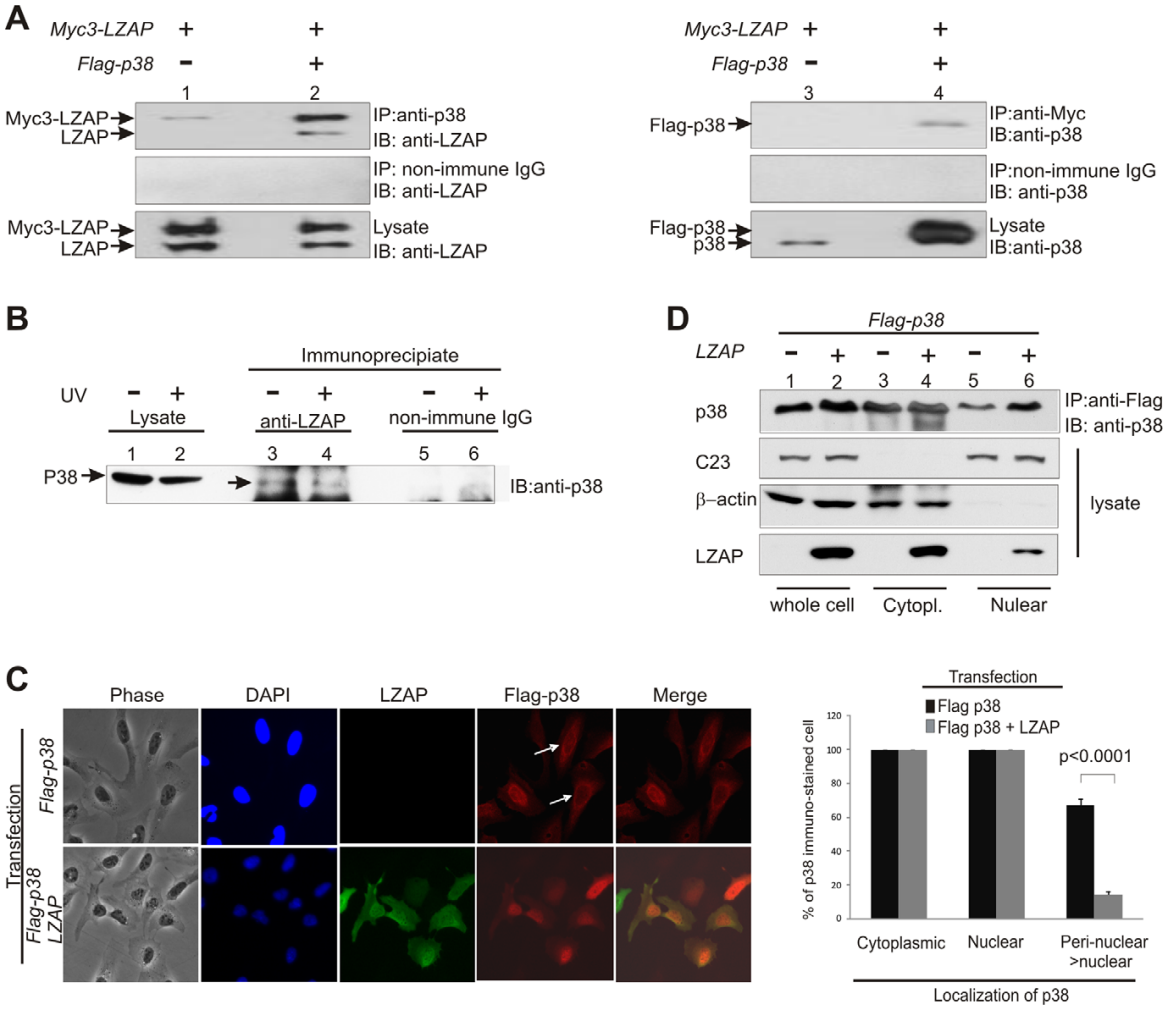
LZAP binds to p38. (A) Ectopically expressed LZAP and p38 mutually co-immunoprecipitate. U20S cells were transfected with indicated plasmids directing expression of tagged LZAP or p38. Immunoprecipitates were prepared using mouse antibodies recognizing Myc (LZAP) or p38, resolved on SDS-PAGE, and immunoblotted by rabbit antibodies recognizing LZAP or p38. Expression of LZAP and p38 was confirmed by immunoblotting (1% of each input lysate was loaded as reference.) Pre-immune IgG was used to control for non-specific immunoprecipitation. (B) Endogenous LZAP binds endogenous p38. Lysates from untransfected MCF7 cells without or with UV irradiation (20 J/m^2^) were immunoprecipitated using LZAP-specific rabbit antiserium or non-immune rabbit IgG, then immunoblotted with antibodies specific to p38 or LZAP as indicated. (C) LZAP co-localizes with p38 and alters p38 subcellular localization. U2OS cells were transfected with plasmids directing expression of Flag-p38 with or without LZAP. Cells were fixed and p38 and LZAP expression and localization determined by indirect immunofluorescence using anti-Flag monoclonal antibody and affinity-purified rabbit anti-LZAP antibody. Cytoplasmic, nuclear and peri-nuclear localization of p38 was determined by direct visualization and quantified based on at least 100 cells from at least 3 independent experiments. The shift of p38 localization from perinuclear region to nucleus was statistically significant (p<0.0001, using unpaired 2 tailed *t* test). (D) Increased p38 abundance in the nuclear following LZAP co-expression. U2OS cells were transfected with plasmids directing expression of Flag-p38 with or without LZAP. Whole cell, cytoplasmic and nuclear cell lysate were prepared, then immunoprecipitates from each lysate were prepared using anti-Flag M2 affinity gel, resolved on SDS-PAGE, and immunoblotted by rabbit p38 antibody. The levels of C23 (nucleolar protein) and β-actin (cytoplasmic protein) were used to monitor the quality of the fractionation and the even loading of samples (1% of each input lysate was loaded as reference.). Expression of LZAP was confirmed by immunoblotting (1% of each input lysate was loaded as reference.), endogenous LZAP could be detected after longer exposure.

To confirm the predicted interaction between LZAP and p38, Myc-tagged LZAP was transiently expressed singly or with Flag-tagged p38 in mammalian U2OS cells before immunoprecipitation. When co-expressed, bands corresponding to Myc-tagged LZAP and untagged LZAP were detected in p38 immunoprecipitates (Fig. 1A, lane 2, top panel). Likewise, p38 was readily detected in LZAP immuno-precipitates (Fig. 1A, lane 4, top panel). Expressed LZAP was also detected in immunoprecipitates of endogenous p38; however, endogenous p38 could not be detected in LZAP immunoprecipitates following LZAP expression (Fig. 1A, lanes 1 and 3, top panels). Expression of proteins was confirmed (bottom panels) and non-immune mouse IgG (middle panels) was used as a control for non-specific immunoprecipitation. To determine if endogenous p38 and LZAP associated in mammalian cells, co-immunoprecipitation of LZAP and p38 was performed using asynchronously growing MCF7 cells, in which LZAP and p38 expression levels are relatively higher compared to U2OS cells (data not shown). p38 was detected in LZAP immunoprecipitates either with or without UV irradiation, but not in precipitates using non-immune rabbit IgG (Fig. 1B, compare lanes 3 and 4 to lanes 5 and 6, the arrow indicates p38). Reciprocal immunoprecipitation using p38-specific antibody did not allow detection of LZAP (data not shown). These data suggest that expressed and endogenous LZAP and p38 exist in a common complex.

To determine if LZAP and p38 co-localized or altered one another's subcellular localization, immunofluorescent staining of LZAP and p38 were performed following single or combined transient expression (Fig. 1C). When expressed without p38, LZAP localizes to both the nucleus and cytoplasm, but is excluded from nucleoli as previously described (data not shown and [3] [2]). In the absence of LZAP, expressed p38 localized to both the cytoplasm and nucleoplasm with more than 60% of cells showing stronger localization to the peri-nuclear region (Fig. 1C, top panel arrows). LZAP localization was not altered by co-expression of p38; however, co-expression of LZAP with p38 resulted in a shift of p38 staining from predominantly peri-nuclear to predominantly nuclear (Fig. 1C, p38 stained panels and graph, p<0.0001).

To confirm the observation that LZAP altered p38 subcellular localization, cellular fractionation was performed on cells expressing p38 with and without LZAP. As expected, both p38 and LZAP localized to both the nuclear and cytoplasmic fractions; however, expression of LZAP increased the amount of p38 detected in the nuclear fraction (Fig. 1D). Fidelity of the nuclear and cytoplasmic fractions was confirmed by expression of nucleolin/C23 and β-actin. Combined, immunofluorescence and cellular fractionation data suggest that LZAP and p38 co-localize and that expression of LZAP increases p38 nuclear localization.

**Figure 2:**
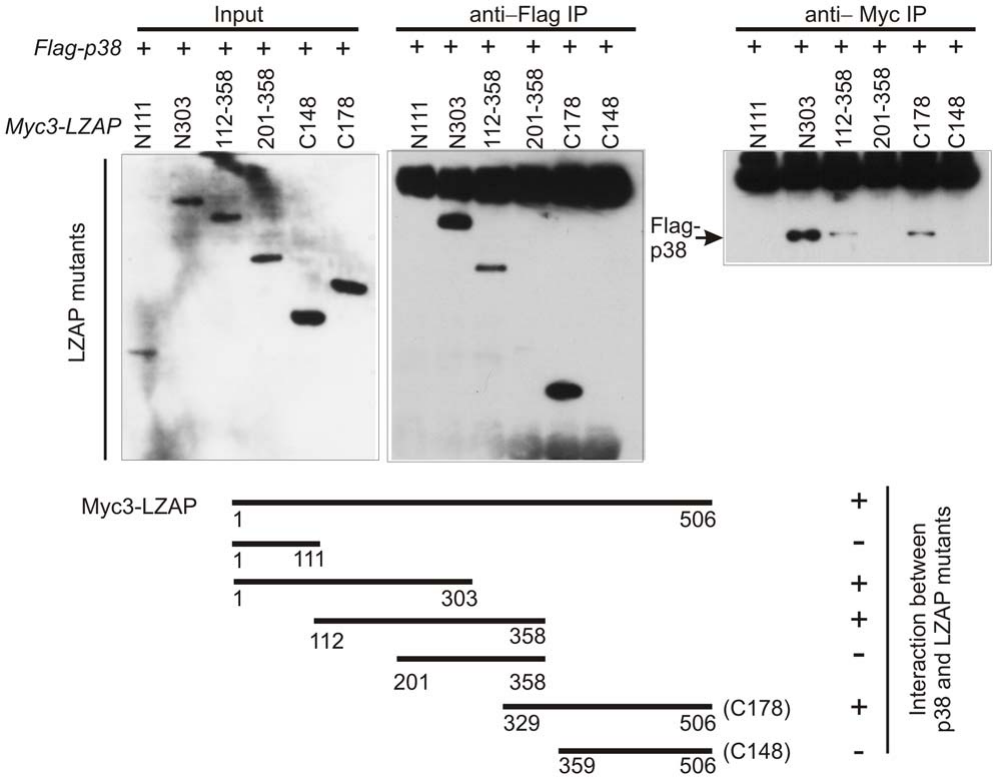
Independent and non-overlapping areas of LZAP are sufficient for association with p38. U2OS cells were transfected with plasmids directing expression of Myc3-tagged truncation mutants of LZAP and full-length Flag-tagged p38. P38 was immunoprecipitated using anti-Flag and LZAP truncation mutants were immunoprecipitated using anti-Myc antibodies before immunoblotting with anti-Myc to detect LZAP truncations or anti-p38. Expression of LZAP truncation mutants was confirmed by immunoblotting and binding activity of LZAP truncation mutants is schematically summarized.

To begin defining regions of LZAP required for p38 interaction, LZAP truncation mutants were co-expressed with full length p38 before co-immunoprecipitation (Fig. 2). Results following either p38 or LZAP immunoprecipitation revealed that an extended LZAP amino terminus region (αα1-303) was sufficient for binding to p38. Within the amino terminal region of LZAP, αα 1-111 was unable to bind p38, suggesting that αα 112-303 were required for this binding. A separate and non-overlapping extended carboxy terminal region of LZAP (αα 329-506) was also sufficient for p38 binding. Truncation of the extended carboxy terminal region abrogated p38 binding suggesting that αα 329-359 of LZAP are required for p38 binding; however, amino acids 329-359 of LZAP were not sufficient for binding to p38 because a central LZAP truncation containing this region (αα 201-358) failed to bind. Within the central region, an LZAP fragment containing residues 112-358 was capable of binding p38 suggesting that a critical domain for p38 binding exists between amino acids 112 and 201 of LZAP (Fig. 2). Results of these p38 binding experiments using LZAP truncations are summarized (Fig. 2).

### LZAP inhibits phosphorylation of p38

**Figure 3:**
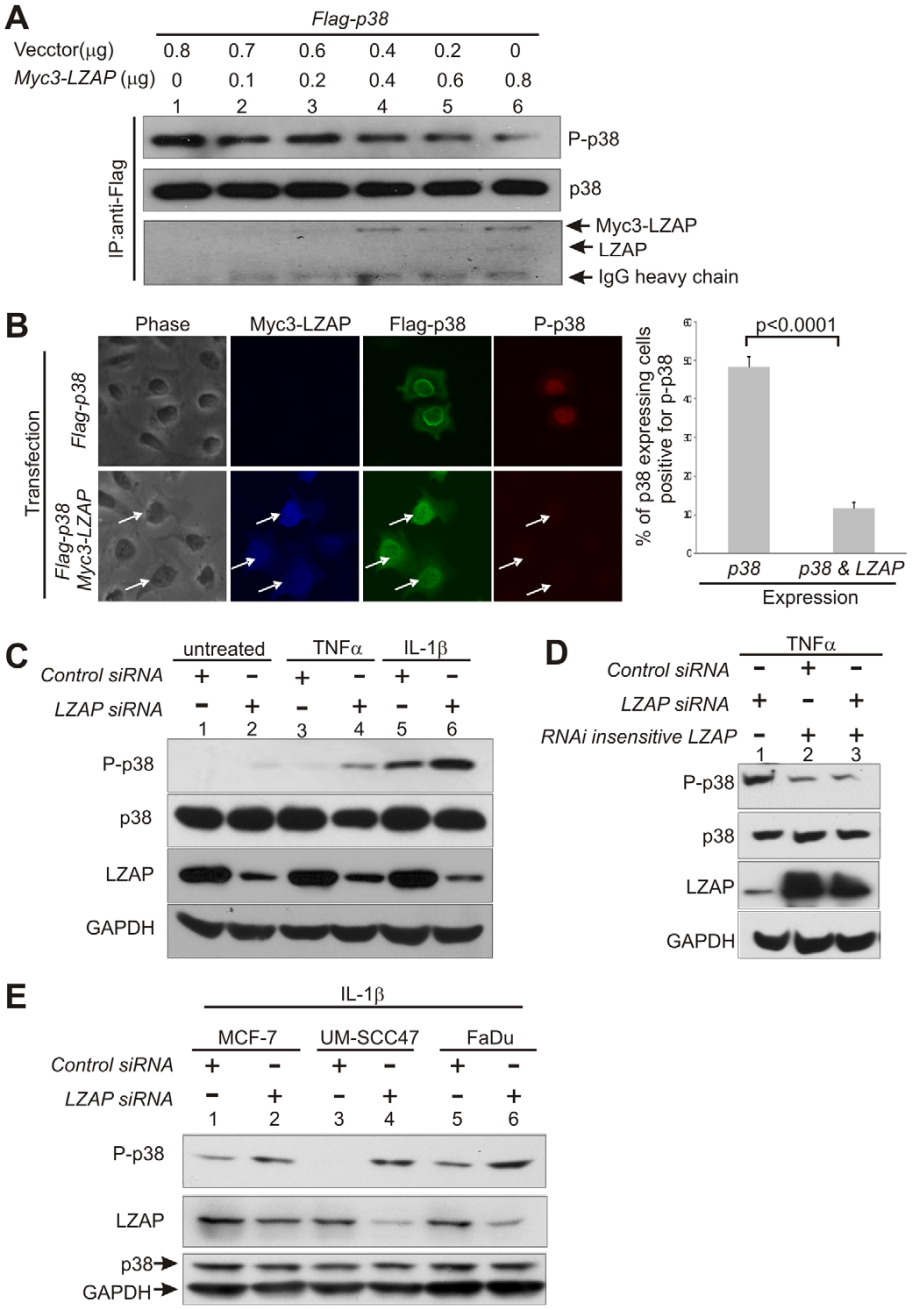
LZAP regulates p38 phosphorylation. (A) LZAP inhibits phosphorylation of p38 at Thr180/Tyr 82. U2OS cells were transfected with plasmids encoding Flag-p38 and with or without increasing amounts of Myc3-LZAP plasmid as indicated. p38 was immunoprecipitated using anti-Flag antibody and p38 phosphorylation at Thr180/Tyr182 determined by immunoblotting. Levels of p38 and LZAP in p38 immunoprecipitates were determined by immunoblotting with antibodies recognizing p38 or LZAP as described [2]). (B) LZAP inhibits accumulation of phosphorylated p38 in the nucleus. U2OS cells were transfected with plasmids directing expression of Flag-p38 with or without Myc3- LZAP. After UV irradiation (20 J/m^2^), cells were triply immunostained with anti-phopho-p38, anti-Flag, and anti-Myc. The fraction of p38 expressing cells with detectable phosphorylated p38 was determined by direct visualization. Expression of LZAP was associated with a significant decrease in detection of phosphorylated p38 in p38 expressing cells ((p<0.0001, using unpaired 2 tailed *t* test). Data are derived from examination of at least 200 cells from at least three independent experiments. (C) Depletion of LZAP increases phosphorylation of p38 in U2OS cells. U2OS cells were transiently transfected with control siRNA or siRNA specific to LZAP. Activating phosphorylation of p38 at Thr180/Tyr182 was determined in untreated cells or in cell treated with TNFα or IL-1β by immunoblotting. Expression of LZAP and total p38 was confirmed and GAPDH was used as a loading control. (D) Depletion of the LZAP protein correlates with p38 activation. Twenty-four hours after transfection with control siRNA or siRNA targeting LZAP, U2OS cells were transfected with plasmid encoding RNAi-insensitive LZAP. Transfected cells were selected with G-418 stimulated using TNFα and p38 phosphorylation determined by immunoblotting. Immunoblotting confirmed expression of p38 and si-RNA insensitive LZAP as described [2]. Immunoblotting of GAPDH served as control. (E) Depletion of LZAP increases phosphorylation of p38 in other cell types. MCF7, UM-SCC47 and FaDu cells were transiently transfected with control siRNA or siRNA specific to LZAP. Activating phosphorylation of p38 at Thr180/Tyr182 was determined in cells treated with IL-1β by immunoblotting. Expression of LZAP and total p38 was confirmed and GAPDH was used as a loading control.

MAPK family members are activated by phosphorylation and upon activation are translocated from the cytoplasm to the nucleus [23], [24], [25]. The dramatic relocalization of p38 from predominantly perinuclear to predominantly nuclear in the presence of LZAP (Fig. 1C and 1D) suggests that LZAP may alter p38 activity. To begin exploring effects of LZAP on p38 activity, total and phosphorylated p38 were detected following transient expression of p38 with or without increasing amounts of Myc-LZAP (Fig. 3A). Expression of LZAP decreased the amount of phospho-p38 detected in a dose dependent fashion, but did not alter total p38 levels. As expected, the amount of LZAP found in p38 immune-complexes increased as LZAP expression increased (Fig. 3A, bottom panel).

Expression of LZAP was associated with increased nuclear p38 levels, but surprisingly, LZAP was also found to decrease total cellular phospho-p38 levels (Fig. 1C, 1D, and 3A). To determine if nuclear p38 was phosphorylated in the presence of LZAP, immunofluorescent staining of p38, phospho-p38, and LZAP was performed following transient expression of Flag-p38 with or without Myc-LZAP and activation of p38 using UV irradiation (20 J/m^2^). Consistent with our previous findings, LZAP expression increased nuclear localization of p38 as indicated by immunofluorescence (Fig. 3B, lower panel green). Despite its ability to increase levels of nuclear p38, LZAP strongly inhibited accumulation of nuclear phospho-p38 following UV irradiation (Fig. 3B, compare upper and lower red panels). Quantification of phospho-p38 results revealed that 48% of p38 expressing cells were positive for nuclear phospho-p38 in the absence of expressed LZAP, compared to only 11% of p38 expressing cells positive for nuclear phospho-p38 in the presence of expressed LZAP (Fig. 3B, p<0.0001). Regardless of LZAP expression, cytoplasmic phospho-38 was not detected. Data represent examination of more than 100 cells from 3 independent experiments. Combined, these data suggest that LZAP inhibits phosphorylation of nuclear p38.

### Depletion of endogenous LZAP activates p38

To determine if endogenous LZAP regulates p38, LZAP was depleted by siRNA and p38 phosphorylation determined. Knockdown of LZAP in U2OS cells did not alter p38 expression; however, loss of LZAP was associated with increased levels of phospho-p38 levels in either the presence or absence of activating cytokines, TNFα and IL-1β (Fig. 3C). LZAP loss following siRNA treatment was confirmed and GAPDH was used as a loading control (Fig. 3C). Off target effects of siRNA were explored by simultaneous siRNA mediated knockdown and expression of LZAP containing silent mutations within the targeting siRNA sequence (RNAi-insensitive LZAP). As described above, cells were treated with TNFα to activate p38 resulting in robust phospho-p38 signal following LZAP knockdown (Fig. 3D, lane 1). Regardless of transfection with siRNA targeting LZAP, expression of RNAi-insensitive LZAP resulted in marked inhibition of phospho-p38 levels (Fig. 3D, lanes 2 and 3). To determine if LZAP activity was cell type specific, p38 phosphorylation after LZAP knockdown and IL-1β stimulation was determined in 1 breast cancer cell line (MCF-7) and in 2 head and neck squamous cell carcinoma lines (UM-SCC47, FaDu).As observed in U2OS cells, siRNA-mediated loss of LZAP was associated with increased p38 phosphorylation (Fig. 3E). Taken together, these data suggest that endogenous LZAP alters p38 phosphorylation both in the presence or absence of activating cytokines.

**Figure 4:**
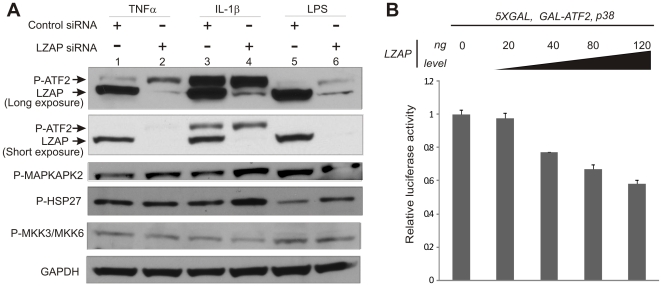
Loss of LZAP increases p38 kinase activity, but does not alter MKK activation. (A) Depletion of LZAP increases phosphorylation of p38 targets, but does not alter phosphorylation of MKK3/MKK6. U2OS cells were transfected with control siRNA or siRNA targeting LZAP before stimulation with TNFα, IL-1β, or LPS. Phosphorylation of direct or indirect p38 targets ATF2, MAPKAPK2, HSP27 and activators of p38, MKK3 and MKK6, was visualized by immunoblotting. LZAP knockdown was confirmed by immunoblotting and expression of GAPDH was used as a loading control. (B) LZAP inhibits transcriptional activity of the p38 target ATF2. U2OS cells were transfected with plasmids directing expression of GAL-ATF2 and p38, with or without increasing amounts of LZAP along with a luciferase reporter containing the GAL DNA binding sequence, as indicated. Firefly luciferase activity was normalized based on renilla luciferase activity and assigned a value of 1 in cells without transfected LZAP. All normalized luciferase assay data are expressed as the mean with the standard error and are the result of the least three independent experiments.

Upstream MAPK kinases (MKK3 or MKK6) activate p38 through direct phosphorylation at thr180 and tyr182 [26]. Once activated, p38 phosphorylates downstream target proteins including: MAPKAPK2 and the transcription factor ATF2. To determine if increased p38 phosphorylation observed upon loss of endogenous LZAP correlates with p38 kinase activity, phosphorylation of p38 target proteins) was measured in the presence or absence of siRNA targeting LZAP (Fig. 4A). After cytokine or LPS stimulation, knockdown of LZAP resulted in increased phosphorylation of p38 targets ATF2 and MAPKAPK2. MAPKAPK2 is itself a kinase that directly phosphorylates HSP27. siRNA-mediated loss of LZAP was associated with increased HSP27 phosphorylation suggesting that a kinase cascade downstream of p38 was activated upon LZAP loss. To begin exploring potential mechanisms of LZAP activity toward p38, the effect of LZAP loss on MKK3 and MKK6 phosphorylation was determined. LZAP knockdown was not associated with increased phosphorylation of upstream p38 kinases, MKK3 or MKK6 (Fig. 4A). Decreased LZAP expression was confirmed by immunoblotting following siRNA treatment (data not shown).

p38-mediated phosphorylation of ATF2 activates ATF2 transcriptional activity [27]. In the presence of cytokines, loss of LZAP expression was associated with increased ATF2 phosphorylation (Fig. 4A), suggesting that LZAP expression may inhibit ATF2 transcriptional activity. To explore this possibility, a luciferase reporter system relying on a chimeric transcription factor construct containing the GAL4 DNA binding domain fused to ATF2 transcriptional activating domain was used as a surrogate for measurement of ATF2 transcriptional activity [28]. In the presence of p38, expression of LZAP resulted in a dose-dependent decrease in ATF2 transcriptional activity (Fig. 4B). Data represent 3 independent experiments. Taken together, these data suggest that phosphorylation and activity of p38 and downstream p38 targets are inhibited by endogenous LZAP and that upstream MKKs are not mediating LZAP activity toward p38.

### LZAP alters Wip1 association with p38

Loss of LZAP did not result in activation of upstream p38-activating kinases MKK3 or MKK6 (Fig. 4A), suggesting that LZAP-mediated inhibition of p38 occurred through alternate mechanisms. Activity and phosphorylation of p38 reflects a balance between upstream activating kinases and inactivating protein phosphatases. Wip1 is a nuclear protein phosphatase that was found to be expressed in response to p53 and to complete a negative feedback loop through inhibition of p53 [29]. In addition to its role in abrogating p53 activity, Wip1 was found to form a physical complex with p38 *in vivo* and to directly dephosphorylate and inactivate p38 [10], [29], [30], [31], [32].

**Figure 5:**
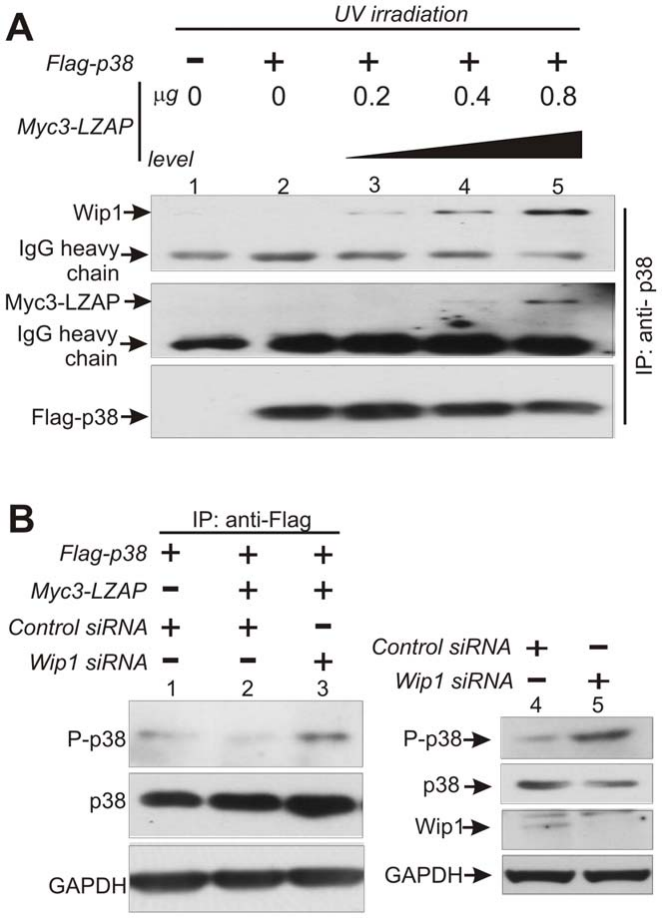
LZAP regulation of p38 phosphorylation involves Wip1. (A) LZAP increases Wip1 association with p38. U2OS cells were transfected with plasmids directing expression of Flag-p38 with or without increasing amounts of myc-LZAP as indicated. Immunoprecipitation of Flag-tagged p38 was followed by immmunoblotting using antibodies specific to Wip1, or p38. (B) Wip1 is required for inhibition of p38 phosphorylation directed by LZAP. U2OS cells were transfected with control siRNA or siRNA targeting Wip1, and after 1 day co-transfected with Flag-p38 with and without Myc3-LZAP, as indicated. Phosphorylation of p38 was visualized by immunoblotting after Flag-p38 immunoprecipitation. Immunoblotting was used to confirm expression of p38 and GAPDH as an indicator of loading.

To determine if the Wip1 phosphatase was involved in LZAP's inhibition of p38, binding of Wip1 to p38 was determined following transient expression of p38 singly or with increasing expression of LZAP. Before p38 immunoprecipitation, cells were UV treated to increase p38 phosphorylation and expression of endogenous Wip1 [10], [33]. Wip1 was not detected in p38 immunoprecipitates in the absence of LZAP (Fig. 5A, lane 2); however, as LZAP expression increased Wip1 association with p38 became detectable and increased concordant with LZAP expression (Fig. 5A, lanes 3–5). In agreement with our earlier findings (Fig. 3A), LZAP expression had no effect on p38 expression; however, increased expression of LZAP correlated with detection of LZAP in p38 immunoprecipitates (Fig. 5A).

Expression of LZAP resulted in increased association between p38 and its direct phosphatase, Wip1, suggesting that decreased phosphorylation and activity of p38 following LZAP expression may be mediated by Wip1. To explore this possibility, phosphorylation of p38 was compared following transient expression of LZAP with or without siRNA mediated inhibition of Wip1 expression. As expected, expression of LZAP resulted in decreased p38 phosphorylation (Fig. 5B, top panel compare lanes 1 and 2). In the presence of LZAP, loss of Wip1 expression restored p38 phosphorylation suggesting that LZAP-mediated inhibition of p38 phosphorylation was at least partially dependent on Wip1 (Fig. 5B). Increased p38 phosphorylation following Wip1 loss in the absence of expressed LZAP has been previously demonstrated [32], [34] and was confirmed in U2OS cells following transfection of siRNA targeting Wip1 (Fig. 5B, right panel).

## Discussion

Based on loss of expression in human HNSCC and cellular effects associated with loss of LZAP such as enhanced invasion, anchorage independent growth, angiogenesis, and growth of xenograft tumors, LZAP has been identified as a putative tumor suppressor [3]. LZAP contains no enzymatic motifs suggesting that its activities may be derived through protein-protein interactions. A remarkable number of molecules that are implicated in tumorigenesis (e.g. ARF, RelA, Chk1, Chk2, and as described here p38) have been found to bind to LZAP suggesting that LZAP activity may be protean [2], [3], [4], [5]. Although identification of binding partners has provided insight into LZAP function, satisfying mechanisms of pleiotropic LZAP activities have been lacking.

Here we report that LZAP bound to the stress activated protein kinase p38, altered p38 subcellular localization and inhibited p38 phosphorylation and activity. Mechanistically, our data suggest that LZAP inhibition of p38 phosphorylation depends on the Wip1 phosphatase, and that in the presence of LZAP, more Wip1 is associated with p38. Conceivably, LZAP may sequester p38 in the nucleus in a complex with Wip1 as a means of p38 inactivation. Alternatively, unphosphorylated nuclear p38 may have unknown activities or may be sequestered in the nucleus so that upon loss of LZAP, rapid activation of p38 could occur through phosphorylation by nuclear kinases.

Interestingly, LZAP was found to bind p38 through two independent and non-overlapping domains, an amino-terminal region (αα 1-303), and a carboxy-terminal region (αα 329-506) of LZAP. Based on gel filtration, LZAP has been reported to exist in large molecular weight complexes and the pattern of independent regions of LZAP binding to its partners, as we observed, has been reported and may be critical for formation of large protein complexes containing LZAP [6]. It is possible that physical association between independent regions of LZAP and its binding partners are integral to LZAP function; however, we and others have found that LZAP can bind to itself suggesting that dimerization or oligomerization of LZAP could explain detection of multiple independent binding sites within LZAP (data not shown and [6]).

The mechanism of LZAP activity toward the growing list of LZAP-associated proteins (LAPs) is not well understood. LZAP has no described enzymatic activity, suggesting that LZAP may exert its effects through association with other proteins. The finding that a portion of LZAP exists in large molecular weight complexes combined with a potential for LZAP to oligomerize lends credence to this argument and further suggests that LZAP may serve to bring together effector proteins. A large portion of LZAP interacting proteins are phosphorylated, and LZAP expression has been associated with decreased phosphorylation of these proteins. These observations led us to explore if LZAP had activity to inhibit p38 upstream kinases or to activate p38 phosphatases. We found that LZAP did not alter kinase activity, as measured by phosphorylation, of MKK3 or MKK6; however, LZAP was found to increase association of p38 with its direct phosphatase Wip1 (Fig. 5A). It is unclear if regulation of phosphatases is a general mechanism of LZAP activity, but it is clear that it is not a universal mechanism since phosphorylation has not been described to play a role in LZAP-mediated ARF activity. It is intriguing that additional described LAPs including RelA, Chk1 and Chk2 are also targets of phosphatases, including Wip1 [31], [35], [36]. We have previously shown that LZAP activates p53 in the absence of ARF raising the possibility that this ARF-independent activity of LZAP may also depend on Wip1 [2].

Depending on cellular context, p38 can mediate opposing cellular responses as an inducer or inhibitor of proliferation and apoptosis [8]. To date, most data highlights LZAP as a tumor suppressor [2], [3], [4], [5], [7], but its role as a p38 regulator imply that LZAP could also have opposing cellular effects or that LZAP inhibition of p38 could be restricted to circumstances where inhibition of p38 suppresses tumor promoting activity.

## Materials and Methods

### Plasmid constructs

The full coding sequence and truncation mutants of LZAP were subcloned into pcDNA3-Myc3 and pET-His expression vectors [2], [3]. Plasmids Flag-p38α and Gal-ATF2 were generous gifts from Dr. Jiahuai Han (The Scripps Research Institute, La Jolla, CA). LZAP that was not a target of siRNA-2(sense strand: 5′-CAAGGTAT**G**TG**G**ACCGAGT) [3] was constructed by introducing the silent mutations G294A and G297A in pCI-Neo-LZAP.

### Antibodies and Reagents

LZAP polyclonal rabbit antibody has been previously described [2]; Mouse monoclonal antibody were purchased as follows: Flag (M2), and anti-Flag M2 affinity gel (Sigma); mouse monoclonal antibodies specific to Myc (9E10), p38 (A-12), rabbit GAPDH, normal mouse and Rabbit IgG, and secondary mouse and rabbit antibodies (Santa Cruz Biotechnology); rabbit polyclonal to p38 (Abcam); Wip1 rabbit polyclonal antibody (Bethyl); flurophore-conjugated secondary antibodies (Jackson ImmunoResearch Laboratories); chicken anti-human Myc-tag polyclonal antibody (Thermo Scientific); and all other antibodies (Cell Signaling). TNFα, IL-1β, and LPS were purchased from PeproTech.

### Cell culture and transfection

Cell lines were maintained at 37°C with 5% CO_2_, in growth media with 10% fetal bovine serum (FBS) (Invitrogen, Carlsbad, CA). Cell lines were obtained from ATCC or collaborators and have been passed in the Yarbrough lab with biannual authentication of identity based on microsatellite analyses of 3 markers (D7S1482A, Mycl1A and DXS981C). Plasmids were transfected using FuGene6 (Roche, Indianapolis, IN) according to the manufacturer's instructions. The total amount of transfected DNA in any single experiment was kept constant by adding control vector (pcDNA3). Small interfering RNA (siRNA) was transfected at 20 nM using Lipofectamine RNAiMAX (Invitrogen, Carlsbad, CA). Control siRNA duplex (nontargeting #1) was purchased from Dharmacon (Dharmacon, Chicago, IL). The LZAP siRNA-2 was previously described [3] with on-TARGETplus modification, 5′-CAAGGTATGTGGACCGAGT (sense strand); the sequence of Wip1 siRNA is: 5′- GGCUUUCUCGCUUGUCACC dTdT [37] purchased from Dharmacon.

### Immunoprecipitation and immunoblotting

Cells were lysed in 0.5%(v/v) Nonidet P40 lysis buffer [38] supplemented with protease inhibitor cocktail (Roche). Total cell extracts were incubated with specific antibodies and precipitated with protein A or G sepharose beads (GE Healthcare) before washing and suspension in Laemmli and gel electrophoresis followed by immunoblotting as described [39]

### Immunofluorescence assay

Briefly, cells were fixed with paraformaldehyde permeabilized with Triton X-100, and blocked with BSA. Target proteins were visualized following incubation with primary antibodies followed by fluorophore secondary antibodies and visualization as described [40].

### Cell fractionation

Cells were scraped in cytosolic lysis buffer (10 mM Tris-HCl [pH 7.5], 100 mM NaCl, 2.5 mM MgCl_2_, and 40 mg/ml digitonin). The lysate was incubated on ice for 5 min and centrifuged (2100 g, 8 min, 4°C), and the supernatant was designated as soluble cytosolic fraction. The pellet was washed with the same buffer before adding RIPA lysis buffer (10 mM Tris-HCl [pH 7.4], 150 mM NaCl, 1% NP-40, 1 mM EDTA, 0.1% SDS, and 1 mM DTT), incubated on ice for 5 min and centrifuged (14,000 rpm, 10 min, 4°C), to obtain the nuclear fraction. Whole cell lysates were prepared using RIPA buffer, as described [41].

### Luciferase reporter assay

ATF2 reporter gene assay was performed using the Dual-Luciferase Reporter Assay System (Promega) as described [3]. Reporter constructs were co-transfected into U2OS cells maintaining equal plasmid amounts. Luciferase activity was measured 24 hours after transfection following the manufacturer's instructions. Luciferase activity was normalized to renilla activity as a control of transfection efficiency.

## Supporting Information

Table S1 **LZAP** **predicted motifs from Scansite.** LZAP protein coding sequence was inputed for motif scan, and chose high stringency criteria to look for all possible binding motifs.
(TIF)
