# Supplementary figures and images for: LZAP Inhibits p38 MAPK (p38) Phosphorylation and Activity by Facilitating p38 Association with the Wild-Type p53 Induced Phosphatase 1 (WIP1)
Source: PLoS One. 2011 Jan 24;6(1):e16427. doi: 10.1371/journal.pone.0016427 (PMC3026010; doi:10.1371/journal.pone.0016427)

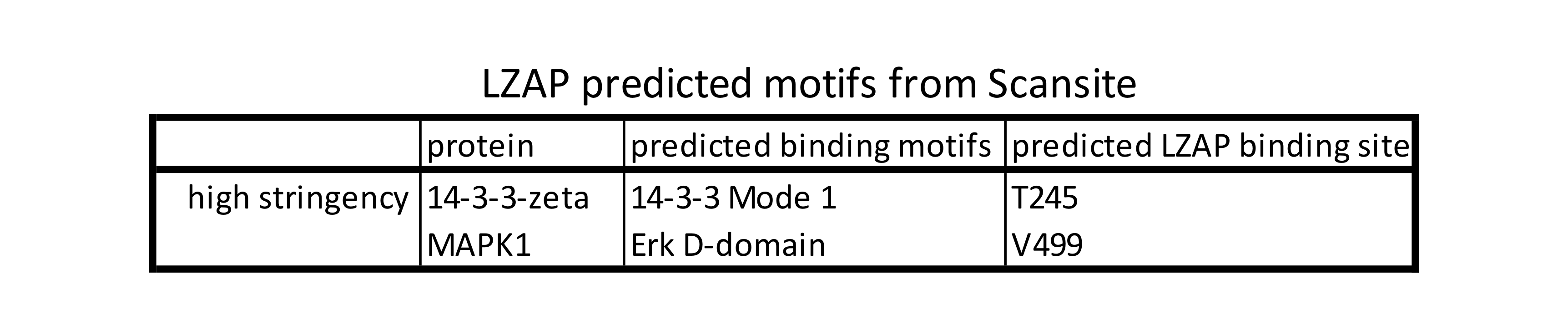

Supplement: Table S1 — LZAP predicted motifs from Scansite. LZAP protein coding sequence was inputed for motif scan, and chose high stringency criteria to look for all possible binding motifs. (TIF) [file pone.0016427.s001.tif]
